# A preliminary study of ^18^F-FES PET/CT in predicting metastatic breast cancer in patients receiving docetaxel or fulvestrant with docetaxel

**DOI:** 10.1038/s41598-017-06903-8

**Published:** 2017-07-26

**Authors:** Chengcheng Gong, Zhongyi Yang, Yifei Sun, Jian Zhang, Chunlei Zheng, Leiping Wang, Yongping Zhang, Jing Xue, Zhifeng Yao, Herong Pan, Biyun Wang, Yingjian Zhang

**Affiliations:** 1 Department of Medical Oncology, Fudan University Shanghai Cancer Center, No.270, Dong’an Road, Xuhui District, Shanghai, China; 2 Department of Oncology, Shanghai Medical College, Fudan University, No.270, Dong’an Road, Xuhui District, Shanghai, China; 3 Department of Nuclear Medicine, Fudan University Shanghai Cancer Center, No.270, Dong’an Road, Xuhui District, Shanghai, China; 4 Center for Biomedical Imaging, Fudan University, No.270, Dong’an Road, Xuhui District, Shanghai, China; 5 Shanghai Engineering Research Center of Molecular Imaging Probes, No.270, Dong’an Road, Xuhui District, Shanghai, 200032 China

## Abstract

The present explorative study was initiated to evaluate the clinical value of ^18^F-FES PET/CT in monitoring the change of estrogen receptor (ER) expression and potential predictive value in metastatic breast cancer patients. Twenty-two pathology-confirmed breast cancer patients were prospectively enrolled and randomly divided into two groups (T: docetaxel, n = 14 and TF: docetaxel + fulvestrant, n = 8). The percentage of patients without disease progression after 12 months (PFS > 12 months) was 62.5% in group TF compared with 21.4% in group T (*P* = 0.08). According to ^18^F-FES PET/CT scans, the SUVmax (maximum standard uptake value) of all the metastatic lesions decreased in group TF after 2 cycles of treatment (6 weeks ± 3 days). However, 6 of 9 patients in group T had at least one lesion with higher post-treatment SUVmax. There was a significant difference in the reduction of ER expression between these two groups (*P* = 0.028). In group TF, the patients with PFS > 12 months had significantly greater SUVmax changes of ^18^F-FES than those with PFS < 12 months (PFS > 12 months: 91.0 ± 12.0% versus PFS < 12 months: 20.7 ± 16.2%; *t* = −4.64, *P* = 0.01). Our preliminary study showed that ^18^F-FES PET/CT, as a noninvasive method to monitor ER expression, could be utilized to predict prognosis based on changes in SUVmax.

## Introduction

Breast cancer, as one of the most common cancers in women, was estimated to account for 15% of newly diagnosed cancers in China in the year of 2015^1^. Estrogen receptor (ER) plays a key role in the development and progression of breast cancers. Approximately 65–70% of women with breast cancer are ER positive (ER+)^2, 3^. Preclinical evidence and clinical evidence have both suggested that ER + breast cancers are less responsive to chemotherapy than ER-negative (ER−) tumors, indicating that ER might interfere with factors determining the sensitivity to chemotherapy^4–7^.

Massive studies have been undertaken to explain the mechanism of ER-mediated drug resistance to find new strategies to reverse resistance. Chemoresistance might be caused by ER itself or by ER modulation of the levels of factors^8–17^. Since the expression of ERα is associated with decreased sensitivity to chemotherapy, inhibition of the ER pathway should naturally reverse ER-mediated chemoresistance. However, previous *in vitro* and clinical data have demonstrated an antagonistic effect between tamoxifen and chemotherapy^12–14^. A possible explanation is that tamoxifen also has estrogen-like agonist activity.

Fulvestrant, which is a new type of selective ER down-regulator, can block ER-mediated transcriptional activity through binding ER and inducing ER degradation^18^. Preclinical evidence has proved that fulvestrant can dramatically reverse resistance to various cytotoxic agents (doxorubicin, paclitaxel, docetaxel, vinorelbine, and 5-fluorouracil), especially with docetaxel, suggesting a novel strategy for reversing ER-mediated chemoresistance^12, 19–22^.

Docetaxel, with a response rate of 30–40%, is considered one of the most effective single agent chemotherapies for breast cancer and was shown to have synergistic effects on inhibiting tumor growth when combined with fulvestrant *in vivo* ^22^. Given the promising preclinical evidence, combination treatment of fulvestrant and chemotherapeutic agents might be beneficial.

With the advent of molecular imaging, positron emission tomography (PET) with ER-targeting radiopharmaceuticals has emerged as a noninvasive method for simultaneously measuring the *in vivo* delivery and binding of estrogen, and thus of ER expression, at multiple sites. Previous studies have successfully validated that ^18^F-FES PET uptake correlates well with immunohistochemical (IHC) scoring for ER^23–28^. Thus, we hypothesized that we could use ^18^F-FES PET to monitor the change in ER during combination treatment, with the potential to predict prognosis.

## Materials and Methods

### Patients

The inclusion criteria were: women between 18 and 70 years old with histologically confirmed hormone receptor (HR)-positive, HER2-negative metastatic breast cancer; an Eastern Cooperative Oncology Group performance status <2; life expectancy ≥3 months; adequate hematologic, hepatic, renal and cardiac function; and at least one measurable site according to the Response Evaluation Criteria in Solid Tumors (RECIST) criteria, version 1.1. Patients included in this study had to have failed previous endocrine therapy (adjuvant therapy or first line therapy for advanced disease) or have rapidly progressive disease needing disease control. Premenopausal women were required to receive ovarian suppression. The enrollment had to occur at least 4 weeks after any previous treatment.

Exclusion criteria were: had previously been treated with fulvestrant, uncontrolled infection or diabetes mellitus, central nervous system metastases, pre-existing ≥ grade 2 peripheral neuropathy, pregnancy or lactation, and any chemotherapy in metastatic settings. Additionally, to avoid pretreatment ^18^F-FES false-negative results, ER antagonists were discontinued for a minimum of 5 weeks before the study.

This study was approved by the Fudan University Shanghai Cancer Center Ethic Committee for Clinical Investigation and all of the methods were performed in accordance with the relevant guidelines and regulations. All of the patients signed written informed consent forms before randomization.

### Treatment and study design

In this single center, open-label, phase II clinical trial (NCT02137083, registration date: 6 May, 2014; details at https://clinicaltrials.gov), patients were randomly assigned to receive docetaxel 75 mg/m^2^ D1 every 21 days (group T) or docetaxel 75 mg/m^2^ D2 every 21 days plus fulvestrant 500 mg D1, 15 and 29 and every 28 days thereafter (group TF). Treatment continued until disease progression, intolerable toxicity, or consent withdrawal.

The primary endpoint of this trial was progression free survival (PFS); secondary endpoints included overall response rate, overall survival and the value of ^18^F-FES PET in monitoring the expression changes of ER. This analysis mainly focused on the clinical value of ^18^F-FES PET; results of other end points were not discussed in this article.

### Synthesis of ^18^F-FES, ^18^F-FDG and quality control

^18^F-FES was synthesized as described by Mori *et al*.^29^ and modified by us, as reported in our previous study^30, 31^. The total preparation time was approximately 100 min, and the corrected radiochemical yield was approximately 40% (at the end of synthesis). After final purification, the radiochemical purity was >99%, and the specific activity was 1–10 Ci/μmol at the time of injection.

^18^F-FDG was produced routinely and automatically by cyclotron (Siemens CTI RDS Eclips ST, Knoxville, Tennessee, USA) using an Explora FDG_4_ module in our center. The radiochemical purity was greater than 95%.

### PET/CT procedure

The patients underwent both ^18^F-FES and ^18^F-FDG PET/CT before and after two cycles of treatment (6 weeks ± 3 days) in our center. The interval between ^18^F-FES and ^18^F-FDG PET/CT was within 7 days.

All of the patients were requested to fast for more than 4 h prior to ^18^F-FES PET/CT scans to eliminate the excretion of ^18^F-FES from the hepatobiliary system and the gastrointestinal tract, which might interfere with image interpretation in the pelvic cavity. An average dose of 222 MBq (6 mCi) of ^18^F-FES was injected over 1–2 minutes. Scanning consisted of a whole-body PET/CT examination (2–3 min per table position) from the proximal thighs to the head and was initiated 1 h after administration of the tracer on a Siemens biograph 16HR PET/CT scanner (Knoxville, Tennessee, USA). The transaxial intrinsic spatial resolution was 4.1 mm (full width at half maximum) in the center of the field of view. PET image data sets were reconstructed iteratively by applying the CT data for attenuation correction, and co-registered images were displayed on a workstation.

Regarding ^18^F-FDG PET/CT scans, all of the subjects fasted at least 6 h, and they had to present blood glucose level less than 10 mmol/L at the time of tracer injection (dosage: 7.4 MBq/kg). Before and after injection, they were kept lying comfortably in a quiet, dimly lit room. The parameters for PET/CT were the same as for ^18^F-FES PET/CT scans.

### Image interpretation

A multimodality computer platform (Syngo, Siemens, Knoxville, Tennessee, USA) was utilized for image review and manipulation. Two experienced board-certified nuclear medicine physicians evaluated the images independently and reached a consensus in cases of discrepancy.

Semi-quantitative analysis of tumor metabolic activity was obtained using the standardized uptake value (SUV) normalized to body weight. Lesions on ^18^F-FES PET/CT scans were identified using paired ^18^F-FDG PET/CT images. When there was no ^18^F-FES uptake was detected in suspicious metastatic lesions, we used other conventional methods (bone scan, ultrasound, CT and MRI) for reference. The maximum SUV (SUVmax) for each metastatic lesion was recorded for further analysis by manually placing an individual region of interest (ROI) on co-registered and fused transaxial PET/CT images. In reference to other ^18^F-FES PET studies and our previous experiences, we used a cut-off value of 1.5 to dichotomize the results into ER positive and negative^32–35^.

The change in SUVmax was defined as the lesion with the largest difference before and after treatment in a patient-based analysis. However, if a patient had higher SUVmax of either ^18^F-FDG or ^18^F-FES after treatment, we used this value subtracted from the pretreatment SUVmax as the change.

Lesions smaller than 1.5 cm were excluded because of partial-volume limitation and resolution restriction. In addition, liver lesions were not included in the ^18^F-FES PET/CT analysis due to their high physiological uptake. In patients with widespread bone metastasis, up to 5 of the largest ^18^F-FES PET lesions corresponding to the most ^18^F-FDG avid lesions, were tabulated for each of 5 areas: skull, thorax (including sternum, scapula, clavicle and ribs), long bones, spine and pelvis.

### Assessments

Radiologic evaluation, including spiral CT or MRI scans, was performed at baseline, every 2 cycles (6 weeks ± 3 days) to confirm treatment efficacy and every 3 months during follow-up until disease progression or death. Tumor responses were confirmed by the investigators according to the RECIST 1.1 criteria. Adverse events (AEs) were monitored throughout the study and were graded according to the National Cancer Institute Common Terminology Criteria for Adverse Events version 4.0.

### Statistical analysis

Data are expressed as the mean ± SD. Normality tests of quantitative data were performed with the Kolmogorov Smirnov two-tailed one sample test.

PFS was defined as the time from random assignment to disease progression or death. Statistical analyses for PFS were performed using the Kaplan-Meier method and were compared between treatment groups using log-rank test.

The change in ^18^F-FES uptake before and after treatment in groups T and TF were compared by Fisher’s exact test. The differences in SUVmax changes between PFS > 12 months and PFS < 12 months in the patients in each group were tested by independent *t* tests. In group TF, for the comparison of pretreatment SUVmax between PFS > 12 months and PFS < 12 months in patients in the lesion-based analysis, we also utilized independent *t* tests. The data were analyzed by the SPSS software packages, version 20.0 (IBM Corporation, Armonk, New York, USA). All of the analyses were two sided. A *P* value less than 0.05 was taken to indicate a statistically difference.

## Results

### Patients and treatment outcomes

From May 2014 to April 2016, 22 women with HR + /HER2- metastatic breast cancer were enrolled, including 8 patients treated with docetaxel and fulvestrant and 14 patients treated with docetaxel monotherapy. The baseline characteristics were well balanced between the two treatment groups (Table 1).

**Table 1:** Patients and tumor characteristics.

Characteristic	TF (n = 8)		T (n = 14)		*P*-value
	No.	%	No.	%	
Age, years					
Median	46		55		
Range	37–68		35–73		
Hormone receptor and Her2 status					
ER positive	8	100	14	100	
PR positive	6	75	14	100	
HER-2 negative	8	100	14	100	
Menopausal status					
Postmenopausal	5	62.5	12	85.7	0.31
Premenopausal	3	37.5	2	14.3	
LHRHa	1	12.5	0	0	
Oothecectomy	2	25.0	1	7.1	
Radical Surgery					
Yes	7	87.5	11	78.6	1.00
No	1	12.5	3	21.4	
Disease-free interval					
<24 m	2	25.0	3	21.4	1.00
>24 m	5	62.5	8	57.1	
No. of metastatic sites					
1	1	12.5	1	7.1	0.91
2	2	25.0	4	28.6	
≥3	5	62.5	9	64.3	
Metastatic sites					
Lung	3	37.5	9	64.3	0.38
Liver	2	25.0	4	28.6	1.00
Bone	5	62.5	7	50	0.68
Visceral disease	5	62.5	12	85.7	0.31

**Figure 1:**
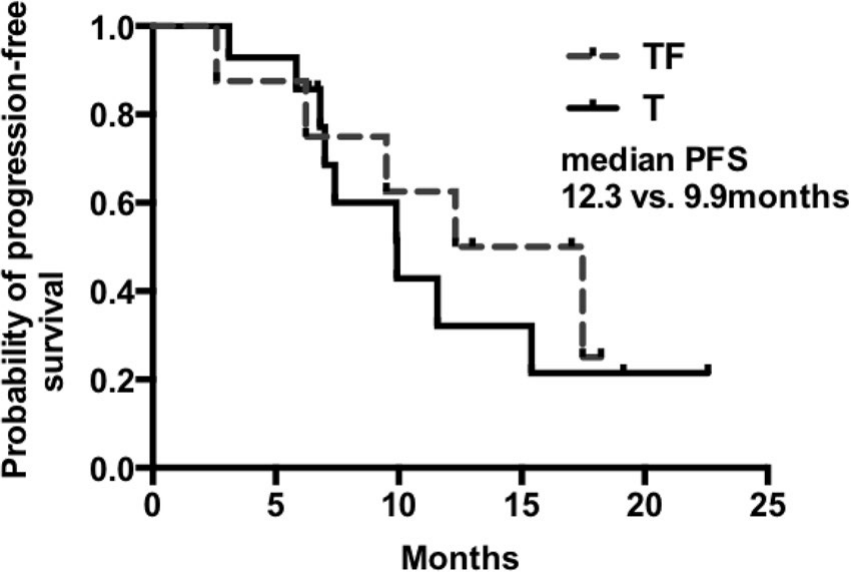
Kaplan-Meier estimates of the progression-free survival of patients treated with docetaxel plus fulvestrant (TF) and with docetaxel monotherapy (T).

This trial was terminated early due to slow enrollment, so that the sample size was not sufficiently powered to detect significant differences of PFS. The primary endpoint, was met by 5 patients (62.5%) in the TF group and 9 patients (64.3%) in the T group by the time of the analysis (Table 2), with a median PFS numerically longer in the TF group than that in the T group (12.3 vs. 9.9 months, Fig. 1). The percentage of patients without disease progression after 12 months (PFS > 12 months) was 62.5% in the combination arm compared with 21.4% in the single-agent docetaxel arm (*P* = 0.08).

**Table 2:** Main clinical outcomes.

Outcomes	TF group*		T group		*P* value
	No.	%	No.	%	
Partial response	5	71.4	11	78.6	
Stable disease	2	28.6	3	21.4	
PFS, months					
Median	12.3		9.9		
95%CI	5.0–20.0		5.8–14.1		
PFS > 12 months	5	62.5	3	21.4	0.08

^*^Included 7 patients with evaluable responses.

### ^18^F-FES and ^18^F-FDG PET/CT Results

5 patients in group T and 2 patients in group TF did not undergo pre- or post-treatment ^18^F-FES PET/CT for various reasons. Therefore, 9 patients in group T and 6 patients in group TF were included for further PET/CT analysis (Table 3). At baseline, a total of 159 metastatic lesions were detected. Lesions were located in lymph nodes (n = 76), bones (n = 32), lungs (n = 22), soft tissue (n = 17), and the liver (n = 12).

**Table 3:** Pre- and post- ^18^F-FES, FDG results and clinical outcomes of each patient. +ongoing.

No.	Group	Tumor sites	lesions (n)	ER expression	Baseline FES SUV	Follow-up FES SUV	%Change FES SUV	Baseline FDG SUV	Follow-up FDG SUV	%Change FDG SUV	PFS (mo)
1	T	liver, lung, lymph nodes, chest wall	21	Increased	1.81	2.98	65	2.88	3.07	7	3.1
2	T	lung	1	Decreased	6	0	−100	2.84	0	−100	9.9
3	T	liver, lung, bone, lymph nodes	17	Decreased	2.76	0	−100	8.11	0	−100	7
4	T	lung, lymph nodes	5	Increased	2.27	2.65	17	3.93	0	−100	9.93
5	T	lung, bone, lymph nodes	17	Decreased	2.33	0	−100	6.78	0	−100	11.57
6	T	lung, lymph nodes	12	Increased	1.51	1.76	17	8.08	0	−100	6.8
7	T	bone, lymph nodes, contralateral breast	13	Increased	2.05	2.2	7	11.14	0	−100	19.13+
8	T	liver, lung, bone, lymph nodes	18	Increased	10.18	17.38	71	5.8	0	−100	15.4
9	T	lymph nodes, pleural membrane	7	Increased	2.49	4.67	86	4.03	0	−100	22.57+
10	TF	lung, lymph nodes	9	Decreased	1.78	1.65	−7	5.16	1.21	−77	2.6
11	TF	liver, bone	7	Decreased	2.37	2.12	−10	2.49	3.65	47	6.23
12	TF	lymph nodes	2	Decreased	2.68	1.46	−45	2.63	3.03	15	9.5
13	TF	bone, lymph nodes, pleural membrane, breast	7	Decreased	9.9	2.72	−73	5.29	3.02	−43	18.23+
14	TF	lymph nodes, breast, chest wall	13	Decreased	1.8	0	−100	15.46	0	−100	17.47
15	TF	lung, bone, pleural membrane	10	Decreased	1.98	0	−100	4.69	0	−100	17.03+

All of these metastatic lesions were FDG avid, with SUVmax values ranging from 1.3 to 15.46. In ^18^F-FES analysis, 145 lesions were included (12 liver lesions and 2 lung lesions adjacent to the liver were excluded; SUVmax = 0.73–20.15). Using a cut-off value of SUVmax = 1.5, 35 lesions were ^18^F-FES negative. Most of the patients (9/15) had both ^18^F-FES-positive and -negative lesions, showing conspicuous heterogeneity of ER expression in these recurrent breast cancer cases.

After 2 cycles of treatment (6 weeks ± 3 days), the ^18^F-FDG uptakes of the majority of lesions decreased (n = 89) or was absent (n = 63); only 7 lesions had higher SUVmax. On ^18^F-FES analysis, 60 lesions showed decreases in ER expression, 59 lesions were absent, and 26 lesions had a higher SUVmax values.

### Fulvestrant reduced ER expression

**Figure 2:**
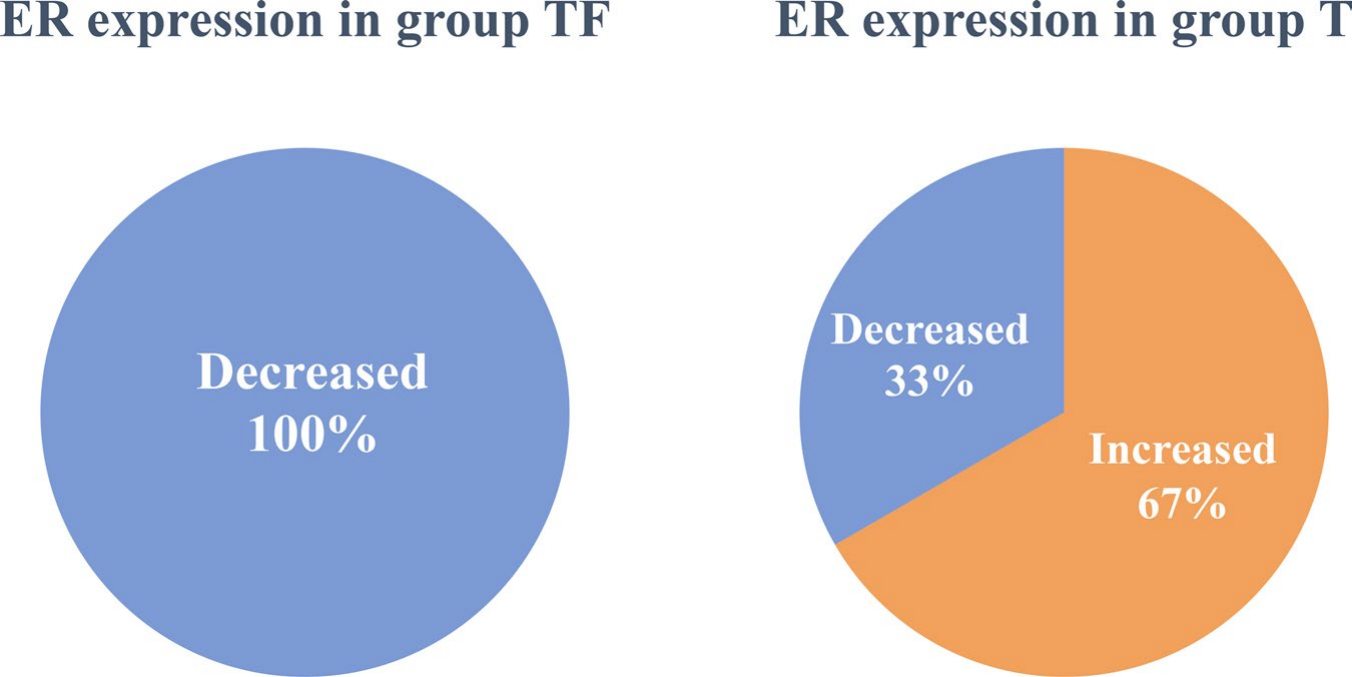
The ER expression changes in patients treated with docetaxel or docetaxel plus fulvestrant.

According to the ^18^F-FES PET/CT scans, the SUVmax values of all of these lesions decreased in group TF after 2 cycle of treatment. However, 6 of 9 patients in group T had at least one lesion with a higher post-treatment SUVmax value (Fig. 2). There was a significant difference in the reduction of ER expression between two groups (*P* = 0.028). The data demonstrated that fulvestrant did reduce the ER expression in metastatic breast cancer patients.

### The change in ER expression showed potential to predict PFS: patient-based analysis

**Figure 3:**
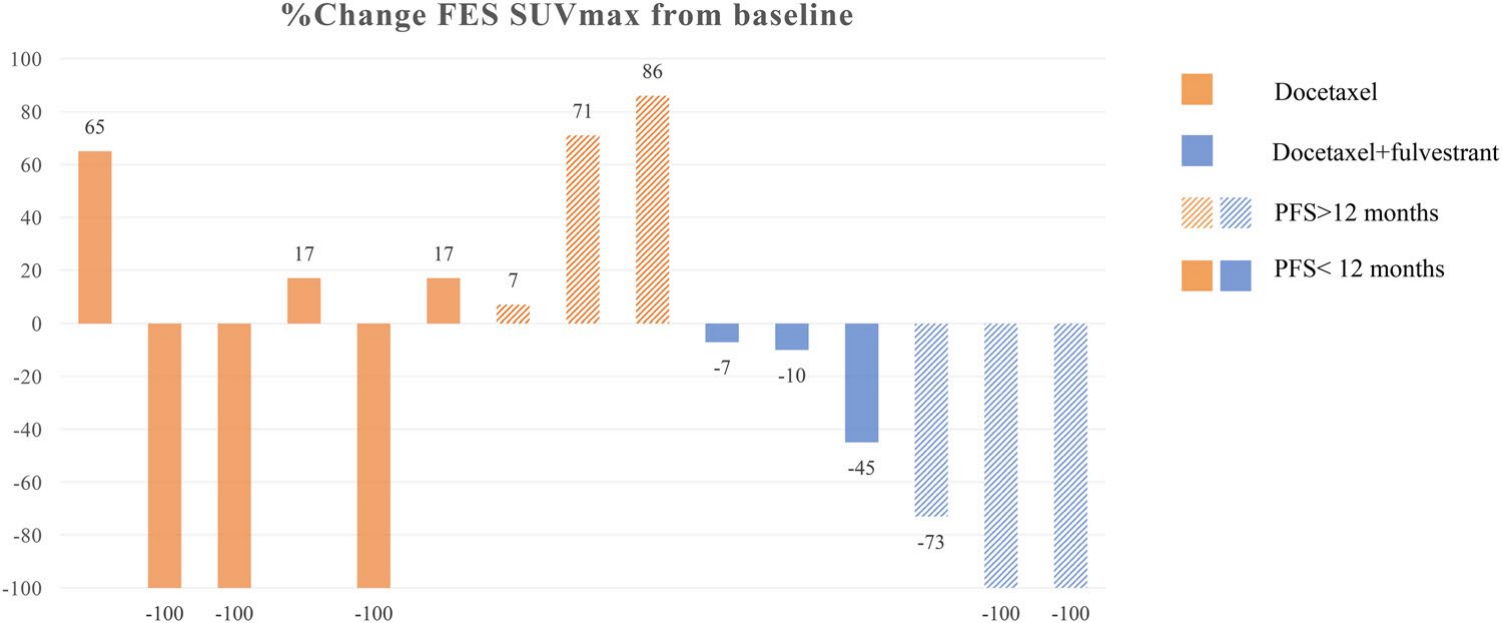
Waterfall plot showing the relative changes in tumor FES uptake in individual patients treated with docetaxel or docetaxel plus fulvestrant on follow-up scans, compared with baseline.

**Figure 4:**
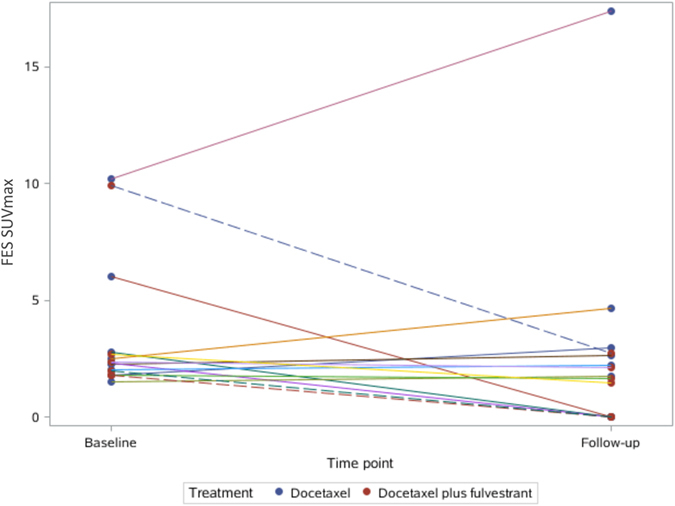
The spaghetti plot of ^18^F-FES changes.

**Figure 5:**
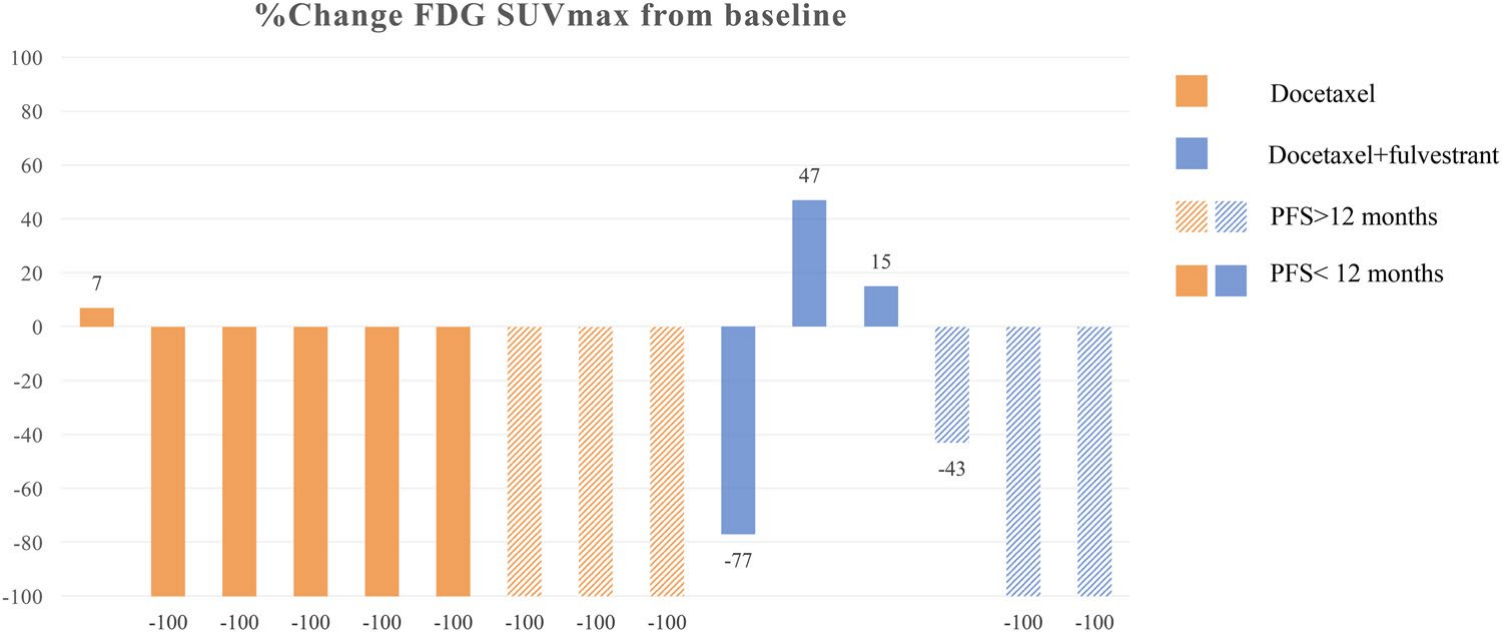
Waterfall plot showing the relative changes in tumor FDG uptake in individual patients treated with docetaxel or docetaxel plus fulvestrant on follow-up scans, compared with baseline.

**Figure 6:**
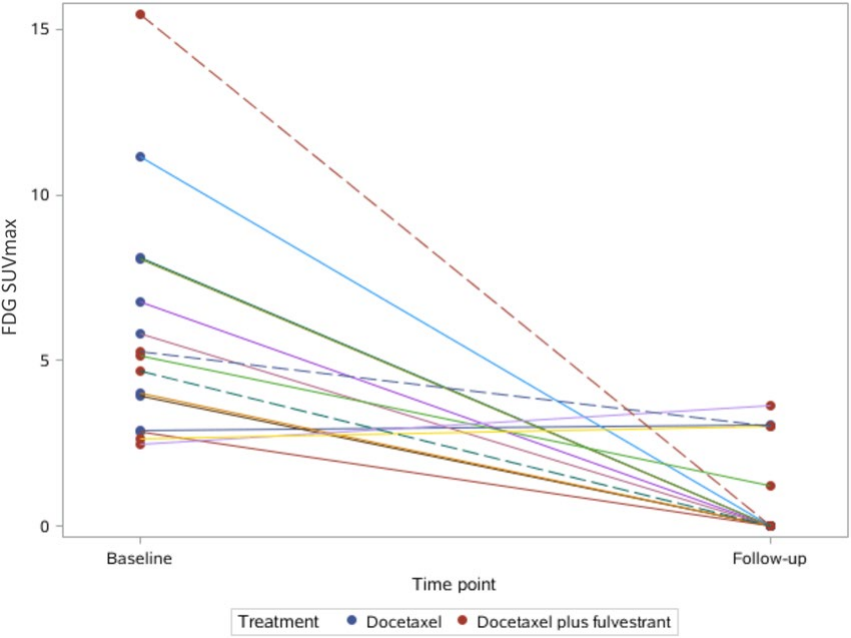
The spaghetti plot of ^18^F-FDG changes.

In group TF, the patients with PFS > 12 months had significantly greater SUVmax changes in ^18^F-FES than those with PFS < 12 months (PFS > 12 months: 91.0 ± 12.0% versus PFS < 12 months: 20.7 ± 16.2%; *t* = -4.64, *P* = 0.01; Figs 3 and 4). However, the change in ^18^F-FDG uptake could not differentiate the patients with better prognosis (PFS > 12 months: 81.0 ± 25.2% versus PFS < 12 months: 5.0 ± 48.0%; *t* = -1.821, *P* = 0.143; Figs 5 and 6).

In group T, the SUVmax changes with neither ^18^F-FES nor ^18^F-FDG showed significant differences between the patients with PFS > 12 months and those with PFS < 12 months (*P* > 0.05).

### Pretreatment ^18^F-FES SUVmax might predict PFS in group TF: lesion-based analysis

**Figure 7:**
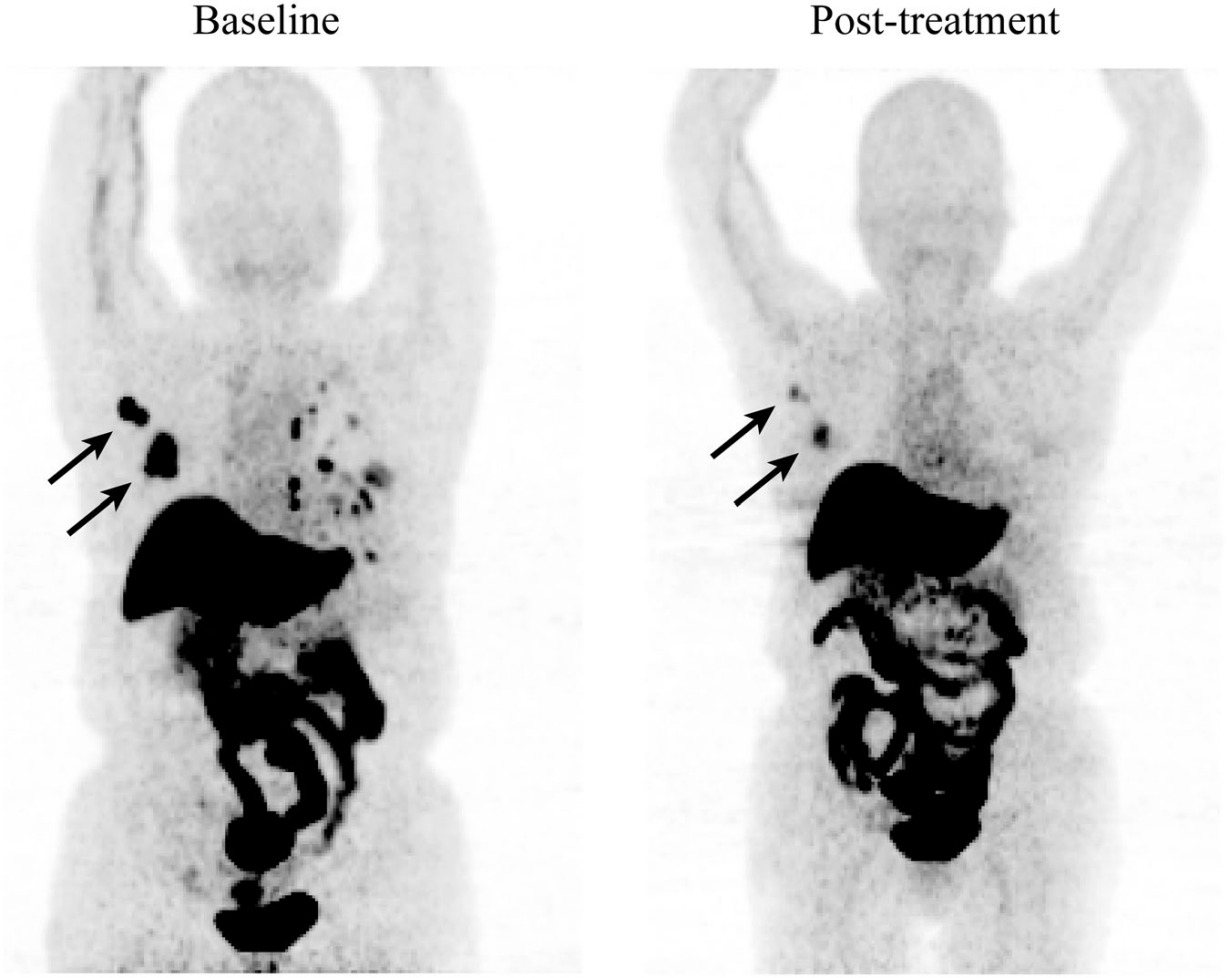
A 68-year-old female breast cancer patient, pretreatment with ^18^F-FES PET/CT (A) showed high uptake in these metastatic lesions (SUVmax = 4.8–20.15). After two cycles of combination treatment (group TF), these lesions had obvious decreases in ^18^F-FES uptake (SUVmax = 2.31–7.26, B). The greatest change was observed in the right axillary lymph node (>70%, arrow). The patient had a PFS > 12 months.

In group TF, there were a total of 48 metastatic lesions detected. Among them, 41 lesions were included for further ^18^F-FES analysis (PFS > 12 months: n = 28; PFS < 12 months: n = 13; 5 liver lesions and 2 lung lesions adjacent to the liver were excluded). The pretreatment ^18^F-FES SUVmax of the metastatic lesions in patients with PFS > 12 months was obviously greater than in patients with PFS < 12 months (PFS > 12 months: 4.1 ± 5.2 versus PFS < 12 months: 1.9 ± 0.5; *t* = 2.175, *P* = 0.038; Fig. 7). Regarding the results mentioned above, the data suggested that fulvestrant might help metastatic breast cancer patients with ER+ lesions to increase their chemosensitivity by reducing ER expression.

On pretreatment ^18^F-FDG SUVmax analysis, however, no significant difference was observed (PFS > 12 months: n = 30, SUVmax = 6.3 ± 3.5 versus PFS < 12 months: n = 18, SUVmax = 5.0 ± 1.7; *t* = 1.678, *P* = 0.1).

## Discussion

As far as we know, this study was the first preliminary study to investigate the feasibility of docetaxel and fulvestrant in HR + /HER2- metastatic breast cancer patients. Our results showed that the addition of fulvestrant to docetaxel improved PFS from 9.9 months to 12.3 months, although no significant difference was observed due to the small sample size. This tendency was consistent with preclinical findings that the combination of fulvestrant and docetaxel had synergistic effect on inhibiting tumor growth^22^.

Because ER plays such important role in chemoresistance, the serial detection of ER during treatment could be useful. Contemporary assessments of ER expression in breast cancer have traditionally conducted *in vitro* assays of biopsied tissue using IHC staining quantitatively or qualitatively. Nevertheless, the presence of ER by IHC does not necessarily guarantee patient benefit from endocrine therapy^36^. Hence, it is far from satisfactory. The reasons could be explained as follows. First, the technique is semi-quantitative. There existed high and consistent rates of both intra- and inter-laboratory variability, and ER scoring also depends on the antibody used and the delay-to-fixation time^37, 38^. It was reported in a systematic review that as much as 20% of all IHC determinations worldwide were inaccurate, according to the American Society of Clinical Oncology and the College of American Pathologists^39^. Second, there was intratumoral heterogeneity of receptor content within the same lesions, as well as variations in ER expression among the primary and metastatic sites^40, 41^. Barry *et al*. suggested that the importance of understanding the role of tumor heterogeneity in measurements of tumor behavior, and they developed approaches and data sets to test the precision of their algorithms^42^. Therefore, we need noninvasive, ER-targeted molecular imaging to observe serial ER expression accurately in clinical practice.

^18^F-FES PET/CT has been evaluated in numerous breast cancer clinical studies as a promising method for assessing *in vivo* ER expression, predicting response (to hormone therapy and adjuvant chemotherapy), evaluating effective ER blockade and assisting in individualized treatment strategy decisions^43–47^. Several previous works, including our own study, have showed that ^18^F-FES PET detected a high occurrence of heterogeneity in recurrent breast cancer patients^48, 49^. Additionally, van Kruchten *et al*.^50^ utilized serial ^18^F-FES to observe tumor estrogen uptake, and it could successfully provide insight into the dose needed for ER antagonists to abolish ER completely. All of the studies mentioned above suggested that ^18^F-FES PET/CT was a useful technique for acquiring ER information sequentially and accurately *in vivo*.

Here, we conducted the first study to use ^18^F-FES PET/CT to observe the changes in ER expression during combination treatment. Our preliminary results were inspiring. According to ^18^F-FES PET/CT scans, the SUVmax values of all of the metastatic lesions decreased in group TF after 2 cycles of treatment. The data demonstrated that fulvestrant did reduce the ER expression in metastatic breast cancer patients. Furthermore, patients with PFS > 12 months had significantly greater SUVmax changes in ^18^F-FES than with PFS < 12 months. All of these findings reflected the potential of ^18^F-FES PET/CT to predict prognosis.

Due to slow enrollment, the trial was terminated early. However, one of the greatest obstacles for enrollment was the high cost of fulvestrant. If we had experimental evidence to select appropriate patients who might benefit from such an expensive drug, it might have been possible for us to recruit patients more easily. Based on the preliminary results with ^18^F-FES PET/CT, we considered that it might be a potential tool for our physicians to make treatment decisions. Further studies could be designed.

In our study, there are several limitations worth mentioning. The first was the small sample. Given the character of the study, we enrolled only 22 patients. We noticed a tendency, but no significant difference in PFS was observed. In addition, only 15 patients underwent both pre- and post-treatment ^18^F-FES and ^18^F-FDG PET/CT. Therefore, we could not demonstrate our results sufficiently. Second, a well-established optimal dose of fulvestrant has not been demonstrated in clinical practice, and it is unknown whether our current dose of fulvestrant, was sufficient for maximal ER downregulation and affording metastatic breast cancer patients the most benefit from the treatment. However, as a noninvasive method, ^18^F-FES PET/CT might guide physicians in choosing the appropriate dose of fulvestrant according to changes in SUVmax in the near future. Third, all of our patients were Chinese; the consequences might be different when compared with other races, thus limiting the generalizability of the results.

## Conclusion

Our preliminary study showed that ^18^F-FES PET/CT, as a noninvasive method to monitor ER expression, could be utilized to observe serial ER regulation during treatment *in vivo* and that it has the potential to predict prognosis; therefore, an individualized treatment strategy could be recommended.
